# Progress in Ophthalmology

**Published:** 1895-07-27

**Authors:** 


### PROGRESS IN OPHTHALMOLOGY.

(*Continued from page 274.*)

**Sub-conjunctival Injections in Eye Disease.** — The whole subject of the administration of mercury by injection has called forth a multitude of papers from various writers, and has shown differences of opinion and of experience that seem almost inexplicable. One of the most important of the long series of contributions was that of Dr. Zossenheim, based upon the experience of Deutschmann's clinic, and followed by an appendix of observation by Deutschmann[18] himself. The method pursued was to use a sublimate solution of 1 per 1,000, and to inject a tenth of the contents of a Pravaz's syringe under the conjunctiva of the globe. The conjunctival surface was carefully disinfected, the syringe itself sterilised, and a bandage was applied for 24 hours after the operation. The latter precaution was soon abandoned as superfluous, and the course finally adopted was to apply a drop of 5 per cent. cocaine solution, to wash the conjunctiva with sublimate solution by a piece of cotton wool, and to use a syringe having a platinum needle pointed with iridium, so that it could be heated to redness in a lamp flame before and after the injection. The puncture was made from six to ten millimetres from the corneal margin. The internal administration of iodide of potassium was carefully avoided. Nineteen cases of parenchymatous keratitis, six of episcleritis, nine of specific plastic iritis, thirteen of non-specific plastic iritis, seven of serous iritis, twenty-nine of choroiditis and irido-choroiditis, and nine of optic nerve atrophy are recorded with sufficient detail. The general conclusion arrived at by the author is that sub-conjunctival injection is the best method of administering mercury in the ocular affections in which it is indicated, and he records no ill consequences from its use. In a few instances a good deal of pain followed the injection, and there was sometimes considerable chemosis and superficial redness, but these effects passed away. The injections were repeated every two or three days, or more frequently, according to the state of the eye and the rate of improvement. Deutschmann, in his appended remarks, relates six cases of septic inflammation after cataract extraction, in which the mischief was arrested by the injections, combined with cauterisation (by Paquelin's or the galvanic cautery) of the wound to prevent further infection, and he concludes his paper in words to this effect: "On the whole, we may certainly consider that the treatment by sub-conjunctival injection is a rapid and trustworthy means of cure in all syphilitic ocular affections, so far, of course, as they are localised in the eye; that it is an important adjuvant to other treatment in the non-syphilitic diseases of the eye; and that it is a sovereign and never-to-be-neglected remedy for acute infective processes, especially for such as are consecutive to operation. In a very large number of cases I have not seen a single instance of any disastrous result."

In opposition to the preceding views Dr. Ludwig Bach[19] has published the results of an experimental research into the nature and treatment of the corneal ulcer produced by inoculation with staphylococcus pyogenes aureus, and contends that the sub-conjunctival injection of sublimate solution is absolutely inoperative. He experimented upon the eyes of rabbits, and having failed to produce any favourable effect upon the disease, he also failed to discover any trace of mercury in the injected eyes themselves or in the connective tissue around them. His contention is that the very small quantity employed is rapidly carried away in the general current of the circulation, and that it neither can nor does exercise any local influence whatever.

The smallness of the dose employed by Zossenheim and the other close followers of Darier, has presented itself as an objection to De Wecker,[20] who, "having no leanings towards the doctrines of Hahnemann," and recollecting the tolerance of the eyes for large injections of saline solutions in cases of retinal detachment, has been injecting half or even the whole of a syringeful of sublimate solution of 1 in 2,000. He says that the therapeutic results have been so marked, by comparison with those produced by less potent injections, that no doubt is admissible as to the speedy efficacy of the large ones. His usual formula is the following: Sublimate, 015 milligr.; salicylate of eserine, 05 centigr.; sterilised distilled water, 30 grammes. If there is iritis, he replaces the eserine by atropine or scopolamine; but in all other cases, he finds that the combination with a myotic materially adds to the beneficial effect of the treatment. Prior to each injection, the cilia are cleansed with a solution of oxycyanuret of mercury, and the conjunctival surface with a 4 per cent. solution of boric acid. After the injection, the eye is closed and dressed antiseptically. The injection is used daily for from one to three days, after which the dose is diminished, and the interval is increased in correspondence with the amount and rate of improvement.

Another contributor to the controversy is Dr. Arnold Marti,[21] who, in a thesis published at Basle, describes the practice which has been pursued in the clinique of Professor Schiess, where the sublimate injections were followed by grave consequences, such as very severe pain and sloughing of the conjunctiva, with obliteration of the sub-conjunctival space; so that it was at last determined to replace sublimate by chloride of sodium, the solution of which was found to be harmless, and was even said to produce all the good effects of mercurial injection. Marti attributes its powers to a quickening of the lymphatic circulation of the eye, with consequent absorption of deleterious matters and of the products of disease. It will be remembered that Rothmund, of Munich, nearly forty years ago, advocated sub-conjunctival injections of chloride of sodium solution as a means of hastening the absorption of corneal nebulæ, and that the practice was for a time largely followed, although it has since fallen into disuse.

**Intravenous Mercurial Injections.**—Dr. Abadie[22] read a paper upon severe syphilitic affections of the eye and their treatment before the Congress of the Société Française d'Ophthalmologie, in which he declared the ordinary forms of administering mercury to be generally insufficient, and expressed his preference for subcutaneous or even for intravenous injections. He had employed the latter in ten or twelve cases daily for more than a year without a single accident, and even without the local abscesses which sometimes follow injections into the connective tissue. He insists upon the necessity of a very strict antisepsis, and uses a syringe which, piston and all, is of glass, and admits of complete sterilisation. The skin is washed with a sublimate solution of 1 in 2,000; but the report of the paper does not give either the formula or the dose of the liquid used for injection. In the discussion which followed, the author mentioned that the intravenous injection of mercury had been first suggested by Baccelli.

[18] Beitrage zür Augenheilkunde, Heft. XV.) [19] Archiv. für Ophthalmologie, Bd. XLI., Abt. 1. [20] Ann. d'Ocul., June, 1895. [21] Ann. d'Ocul., Jan., 1895. [22] Ann. d'Ocul , May, 1895.

